# Supplementary material for: A curvilinear coordinate flatmap for visualizing hippocampal structure and development
Source: Imaging Neurosci (Camb). 2026 Nov 4;4:IMAG.a.1429. doi: 10.1162/IMAG.a.1429 (PMC13736831; doi:10.1162/IMAG.a.1429)
Supplement: Supplementary file 1 [file IMAG.a.1429_supp.pdf]

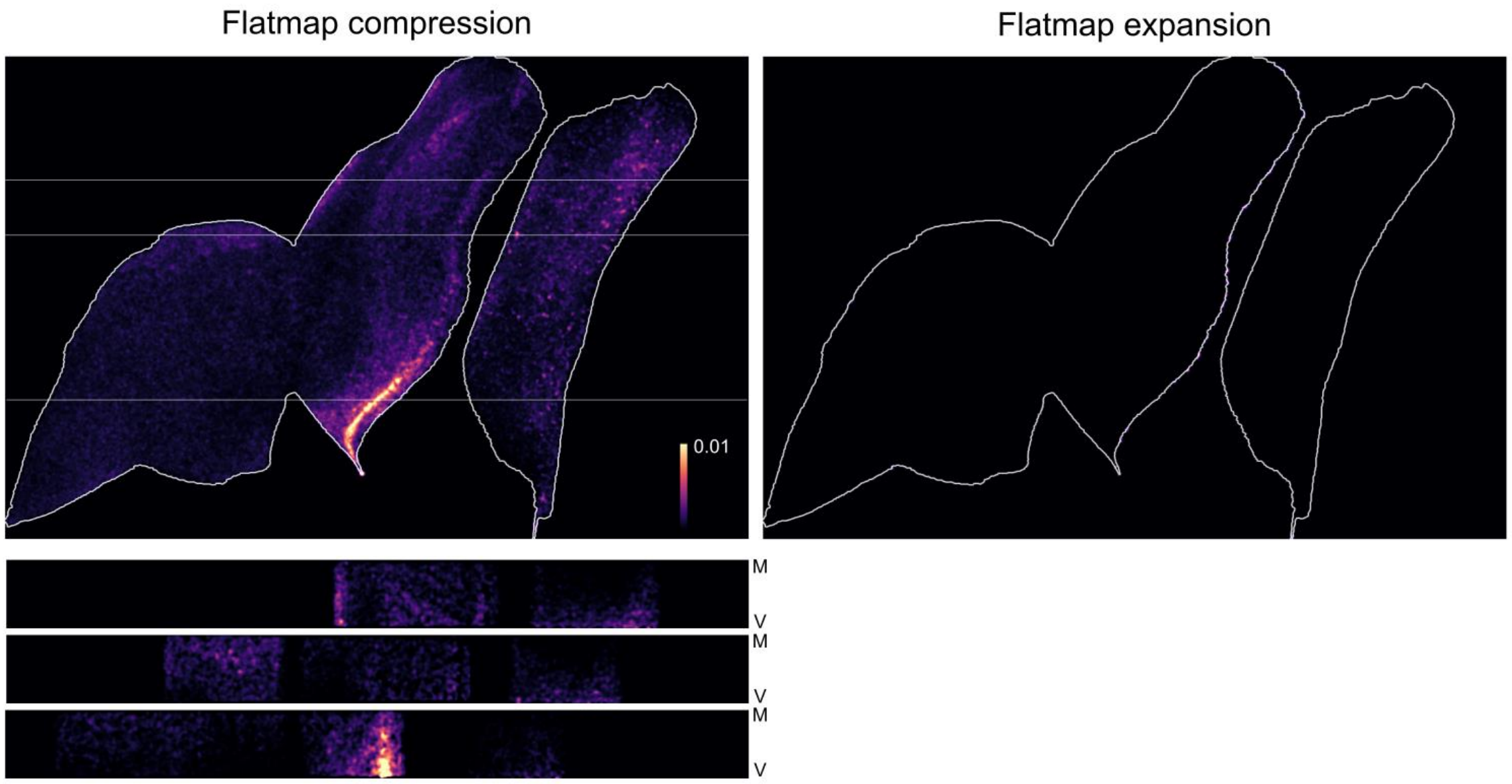

Figure S1
Maximum projection flatmaps showing log-transformed Jacobian determinant values, split by compressed (left) and expanded (right) regions relative to CCFv3. Heatmaps show local log-Jacobian in flatmap (top) and in cross-section along meningeal-ventricular axis (bottom). Horizontal lines show section locations.

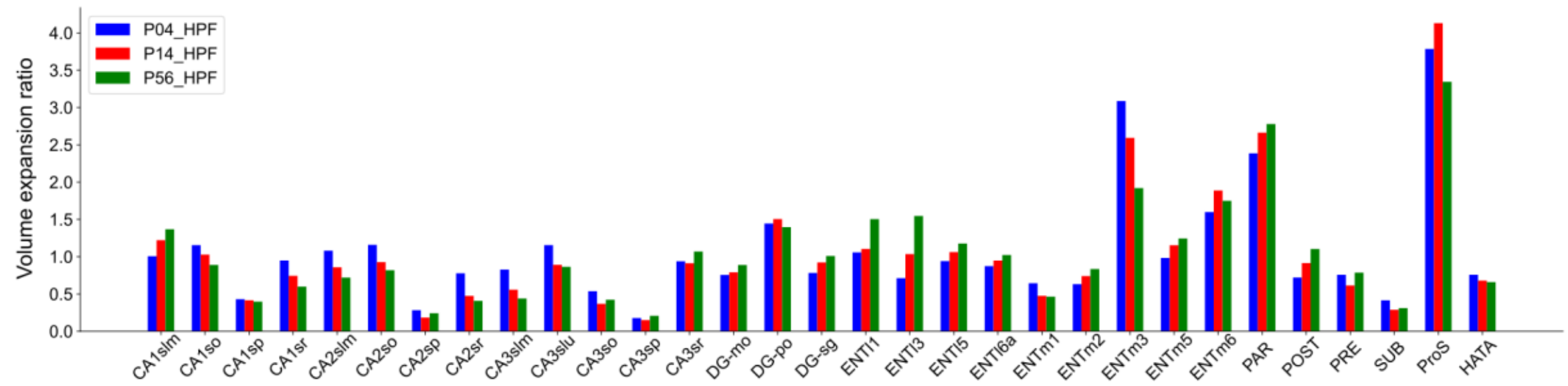

Figure S2

Ratio of flatmap to CCF volume for each hippocampal subregion at P4 (blue), P14 (red), and P56 (green), quantifying nonlinear deformation linked to structural curvature.

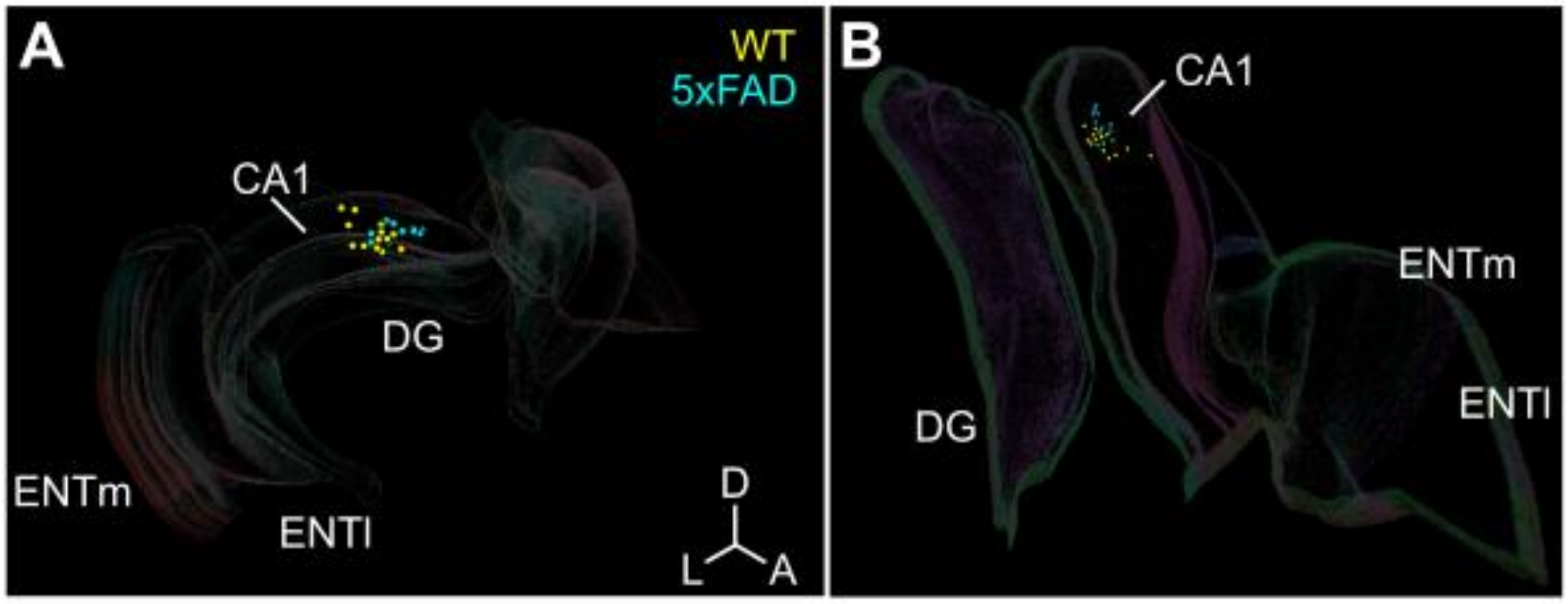

Figure S3
Starter cell distribution for WT (yellow) and 5xFAD (cyan) examples shown in (**A**) CCF coordinate space and (**B**) curvilinear coordinate space. Outlines show major hippocampal divisions.
